# Paeoniflorin Inhibits Hepatocyte Growth Factor- (HGF-) Induced Migration and Invasion and Actin Rearrangement via Suppression of c-Met-Mediated RhoA/ROCK Signaling in Glioblastoma

**DOI:** 10.1155/2019/9053295

**Published:** 2019-02-11

**Authors:** Guoyong Yu, Zhaotao Wang, Shulian Zeng, Sisi Liu, Chunping Zhu, Ruxiang Xu, Ru-en Liu

**Affiliations:** ^1^Department of Neurosurgery, Peking University People's Hospital, Peking University, Beijing, China; ^2^Department of Neurosurgery, The Second Affiliated Hospital of Guangzhou Medical University, Guangzhou, China; ^3^Jiangxi provincial People's Hospital Affiliated to Nanchang University, Nanchang 330006, Jiangxi, China; ^4^Bayi Brain Hospital, General Army Hospital, China

## Abstract

Paeoniflorin (PF), as one of the important valid natural compounds of the total glucosides of peony, has displayed a potential effect in cancer prevention and treatment. Aggressive migration and invasion, as an important process, can contribute to tumor progression through infiltrating the surround normal tissue. Actin cytoskeleton rearrangement plays a key role in cells migration and invasion, involving multiple signal pathways. HGF/c-Met signal, as an important couple of oncoprotein, has been demonstrated to regulate actin cytoskeleton rearrangement. In our study, we aim to explore whether paeoniflorin can inhibit migration and invasion and actin cytoskeleton rearrangement via regulation of HGF/c-Met/RhoA/ROCK signal. Various approaches were applied to demonstrate the mechanism of paeoniflorin-mediated anticancer effect, including cell wound healing assay, invasion assay, immunofluorescence staining and transfection, and western blotting. We observed that paeoniflorin inhibited HGF-induced migration and invasion and actin cytoskeleton rearrangement in glioblastoma cells. Furthermore, the inhibition of HGF-induced migration and invasion and actin cytoskeleton rearrangement involved c-Met-mediated RhoA/ROCK signaling in glioblastoma. Thus, our study proved that paeoniflorin could inhibit migration and invasion and actin cytoskeleton rearrangement through inhibition of HGF/c-Met/RhoA/ROCK signaling in glioblastoma, suggesting that paeoniflorin might be a candidate compound to treat glioblastoma.

## 1. Introduction

Glioblastoma (GMB), as the most common brain cancer in central nervous system, has the most malignant degree. Though we have taken multiple measures, such as radiotherapy, chemotherapy, surgery, or these combined, the median survival time of those who diagnosed with glioblastoma is still not more than 18 months [1–3]. Thus, it is impending to find a new approach to treat GMB. Up to now, more and more natural compounds showed the anticancer activity [4–6]. Therefore, natural products could be thought as potential new antitumor agents to cure GMB.

Paeoniflorin, a polyphenolic natural product, has displayed anticancer activity in a variety of cancer, including breast cancer, pancreatic cancer, gastric cancer, and hepatocellular carcinoma, through inhibiting proliferation, inducing apoptosis, and arresting cell cycle [7–10]. It has been reported that paeoniflorin could induce human pancreatic cancer cell apoptosis [11]. Similarly, Wang et al. showed that paeoniflorin suppresses cell growth and induces apoptosis in multiple myeloma cells [12]. Also, Li et al. reported that paeoniflorin restrains cell growth and promotes apoptosis in human glioma cells [13]. Though, some research has reported that paeoniflorin could inhibit migration and invasion in multiple kinds of cancer cells. For instance, paeoniflorin could induce suppression of invasion in breast cancer cells via inhibition of Notch-1 signaling [14]. Additionally, paeoniflorin could prevent metastasis in hepatocellular carcinoma cells [15]. However, whether paeoniflorin can inhibit migration and invasion in GBM as well as the underlying mechanism is not clear.

Migration and invasion contribute to cancer progression, though the distant metastasis of glioblastoma hardly occurs; it also infiltrates into adjacent normal brain tissue to cause a series of serious consequences [16]. Moreover, the infiltrative growth because of migration and invasion leads to the blurring boundary, which makes it difficult to remove the glioblastoma completely. Meanwhile, migration and invasion involve multiple processes; among them, the actin cytoskeleton dynamic equilibrium is an important one. The actin microfilament system has been considered the engine of cellular migration and invasion [17, 18]. Destroying the steady state of actin cytoskeleton could be an effective approach to inhibit migration and invasion. Up to now, it has not been reported that paeoniflorin can affect actin cytoskeleton arrangement.

HGF/c-Met signal, as a couple of ligand and receptor, plays a major role in progression among multiple cancer types [19–21]. Moreover, the HGF/c-Met has been demonstrated that it highly expressed in glioblastoma and can facilitated glioblastoma malignant phenotype, such as promoting proliferation, antiapoptosis, strengthening migration, and invasion [22–24]. Therefore, some efforts targeting HGF/c-Met have been took to cure the glioblastoma. And that HGF/c-Met has proved that it could affect actin cytoskeleton rearrangement involving the regulation RhoA signal [22–24]. RhoA, as a member of small GTPase protein of Rho family, is primarily associated with actin cytoskeleton regulation. In our study, we explored the effects of paeoniflorin on actin cytoskeleton and deeply investigated whether the process involves the HGF/c-met-mediated RhoA regulation.

The present study was to explore the potential effects of paeoniflorin on HGF-mediated migration, invasion, and actin cytoskeleton rearrangement as well as the underlying mechanism in glioblastoma. In this study, paeoniflorin represses HGF-induced migration, invasion, and actin cytoskeleton rearrangement and this effect involved the suppression of the c-Met-mediated RhoA/ROCK signaling.

## 2. Materials and Methods

### 2.1. Chemicals, Reagents and Antibodies

Paeoniflorin was purchased from Abcam (Beverly, MA) and was dissolved in saline; then it is kept at 4°C. Dulbecco's modified Eagle's medium (DMEM) and fetal bovine serum (FBS) were obtained from Gibco (Grand Island, USA). c-Met inhibitor (SU11274) was purchased from Selleck Chemicals (Houston, TX). Antibodies against c-Met, phospho-c-Met (Y1230/34/35), ROCK1, phospho-limk1(T508), and limk1 were purchased from Abcam (Beverly, MA). Antibodies against GAPDH were purchased from Zhongshangjinqiao Science and Technology Ltd. (Beijing, China). Recombinant human HGF protein was purchased from R&D Systems (Minneapolis, USA). The Upstate® Rho Activation Assay Kit was obtained from EMD Millipore (Lake Placid, USA). Phalloidin was purchased from Abcam (Beverly, MA).

### 2.2. Cell Culture

The cell lines T98G, U251, HA1800, and HEB were obtained from Chinese Academy of Medical Sciences (Beijing, China). These cell lines were cultivated in DMEM contained with 10% FBS in a humidified incubator containing 5% CO2 at 37°C.

### 2.3. Cell Viability Assay

5000 cells/well were planted in the 96-well plate and incubated with different dose of paeoniflorin for 24 hours. Then, 10 *μ*l CCK-8 solution was added to each well and treated for one hour at 37°C. The solution was detected by the microplate reader.

### 2.4. Wound Healing Assay

A wound-healing assay was used to compare the migratory ability of glioblastoma cells in control and experiment groups. The assay was performed with T98G and U251 as described previously [25].

### 2.5. Cell Invasion Assay

The transwell system for assay of cell invasion was obtained from Corning (Corning, USA). The assay was performed with T98G and U251 as described previously [25].

### 2.6. Immunofluorescence

For immunofluorescence, cells were grown on glass coverslips in 12-well plate. After treatment with indicated concentration paeoniflorin for 24h, the cells were fixed with 4% formaldehyde for 15 minutes at room temperature and washed in PBS for 3×5 minutes. Then 1× Phalloidin-iFluor 488 was added and incubated 90 minutes at RT. After being washed in PBS for 3×5 minutes, coverslips were mounted on glass slides using mounting medium (DAPI Fluoromount-G, Thermo Fisher Scientific, USA). At last, the images were obtained with the Laser Scanning Confocal Microscope (Leica, Germany).

### 2.7. Transfection

GBM cell lines were transfected with plasmid carrying c-Met or empty plasmid vector (GeneCopoeia, Maryland Rockville, USA) using lipofectamine 3000 following the instruction's protocol. Then the cells were used in the following experiments.

### 2.8. Measurement of RhoA Activity

RhoA activity was measured using a pull-down assay (Upstate® Rho Activation Assay Kit) according to the manufacturer instructions.

### 2.9. Western Blotting

Western blots were used with glioblastoma cell lysates and performed as described previously [25].

### 2.10. Statistical Analysis

The data are displayed as the mean ± standard error from at least three independent experiments. The similar methods were used for statistical analysis of in vitro and in vivo data described previously [25].

## 3. Results

### 3.1. Paeoniflorin Inhibited Glioblastoma Cells Growth

**Figure 1:**
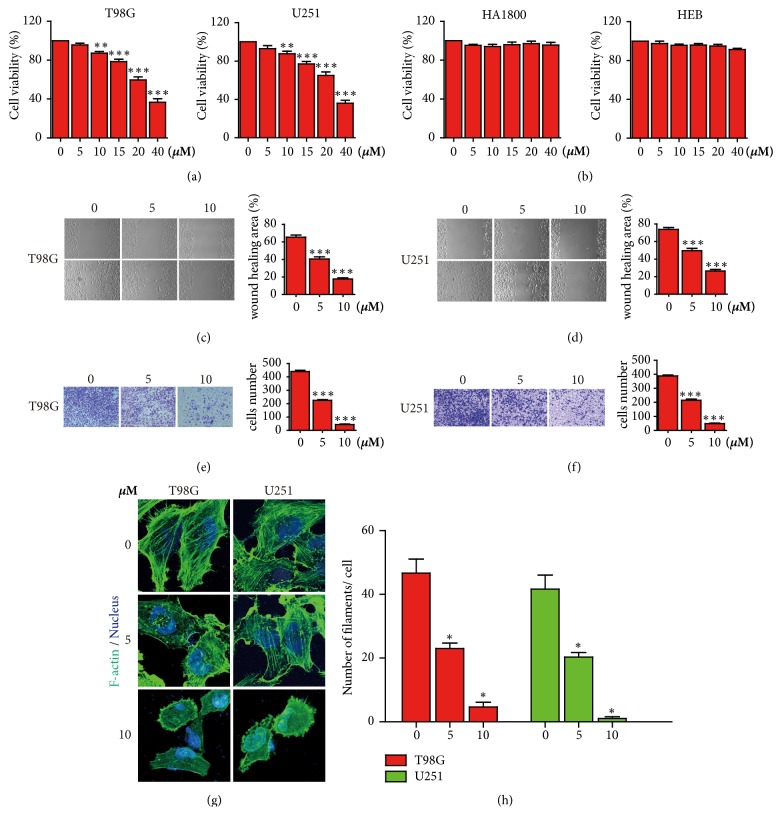
*Effects of paeoniflorin on proliferation, migration and invasion, and actin cytoskeleton arrangement in T98 and U251 cells*. (a-b) cells were incubated with the indicated concentrations of paeoniflorin for 24 hours before CCK-8 assay. (c, d) cells were incubated with 0*μ*M, 5*μ*M or 10*μ*M proliferation after the wounds were scratched. Then representative images of wound healing were acquired after 0 or 24 hours. (e, f) The transwell invasion assay. Cells were treated with the indicated dose of paeoniflorin for 24 hours. Then representative picture was taken. The stained cells were counted. Each represents at least three independent experiments. (g, h) Confocal sections of T98 and U251 cells stained with FITC-phalloidin after indicated concentration paeoniflorin treatment and the quantitative analysis of the number of actin filaments. All tests were performed in triplicate and presented as mean ± standard error. *∗∗*P<0.01, *∗∗∗*P<0.001, compared with control (0 *μ*M).

To examine whether paeoniflorin treatment inhibits cell growth in glioblastoma cells, CCK-8 assay was applied to detect the growth viability in U251, T98G, HA1800, and HEB cells incubated with different dose of paeoniflorin for 24h. It showed that paeoniflorin significantly inhibited cell growth in a concentration-dependent manner in U251 and T98G cells, but not in HA1800 or HEB (the two normal astrocytes) (Figures 1(a) and 1(b)). Moreover, when treated with paeoniflorin in concentrations of 5*μ*M and 10*μ*M, it displayed the little inhibited ability. Therefore, we selected 5*μ*M and 10*μ*M of paeoniflorin in the subsequent experiments.

### 3.2. Paeoniflorin Suppressed Glioblastoma Cells Migration, Invasion, and Induced Actin Rearrangement

Wound healing assay and transwell assay were conducted to detect the effects of paeoniflorin on migration and invasion in glioblastoma cells. Compared with control group (0 *μ*M), the wound healing assay displayed that paeoniflorin significantly inhibited the migration of U251 and T98G cells after 24 hours treatment paeoniflorin (Figures 1(c) and 1(d)). And transwell assay further validated that paeoniflorin treatment suppressed the invasion of glioblastoma cells transit from the matrigel-coated membrane (Figures 1(e) and 1(f)). Moreover, we found that paeoniflorin suppressed cell migration and invasion of U251 and T98G cells in a dose-dependent manner (Figures 1(c)-1(f)). Therefore, these results suggest that paeoniflorin has an antimigration and invasion activation in glioblastoma cells.

Cell migration and invasion implies changes in F-actin cytoskeleton. Therefore, the CytoPainter Phalloidin-iFluor 488 (Abcam, USA) was used to examine the cytoskeletal F-actin pattern. In normal, the F-actin arranges orderly and continuously in cells. Compared with the control group, after treatment with paeoniflorin 5*μ*M for 24 hours in U251 and T98G, the normal actin filaments structure began to become disordered and sparse. Moreover, when the concentration is up to 10*μ*M, the normal actin filaments structure almost disappeared and the number actin filaments was reduced, at the same time, the morphology of U251 and T98G began to change that turned from spindle-shape to round-shape (Figures 1(g) and 1(h)). These results suggest that paeoniflorin induces the actin rearrangement in U251 and T98G.

### 3.3. Paeoniflorin Downregulated RhoA Activation in Glioblastoma Cells

**Figure 2:**
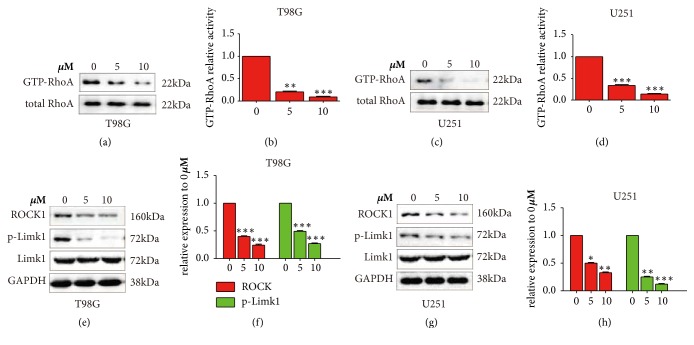
*Paeoniflorin suppressed RhoA/ROCK signaling*. (a-d) the GTP-RhoA activity after different dose of paeoniflorin incubation for 24 hours in T98G and U251 cells. (e-h) the protein expression of ROCK1 and p-Limk1 after different concentration of paeoniflorin treatment for 24 hours in T98G and U251 cells. All tests were performed in triplicate and presented as mean ± standard error. *∗∗*P<0.01, *∗∗∗*P<0.001, compared with control (0 *μ*M).

Rho-GTPase plays an important role in the actin cytoskeleton rearrangements. To determine whether paeoniflorin may regulate the Rho-GTPase in glioblastoma cells, we examined the RhoA activity. As shown in Figures 2(a)-2(d), active form of RhoA, the GTP-RhoA, was significantly decreased after paeoniflorin treatment in U251 and T98G cells. Furthermore, to confirm the effect of paeoniflorin on RhoA signaling, the downstream RhoA/Rho-associated kinase (ROCK) and the phosphorylation-LIM kinase-1(Limk1), a target of ROCK1, were examined. The results showed that paeoniflorin significantly downregulated ROCK1 and Limk1 in U251 and U87 cells in a dose-dependent way (Figures 2(e)-2(h)). These results suggest paeoniflorin downregulated RhoA activation in glioblastoma cells.

### 3.4. Paeoniflorin Inhibited HGF-Mediated Glioblastoma Cells Migration and Invasion and Leaded to Actin Rearrangement

**Figure 3:**
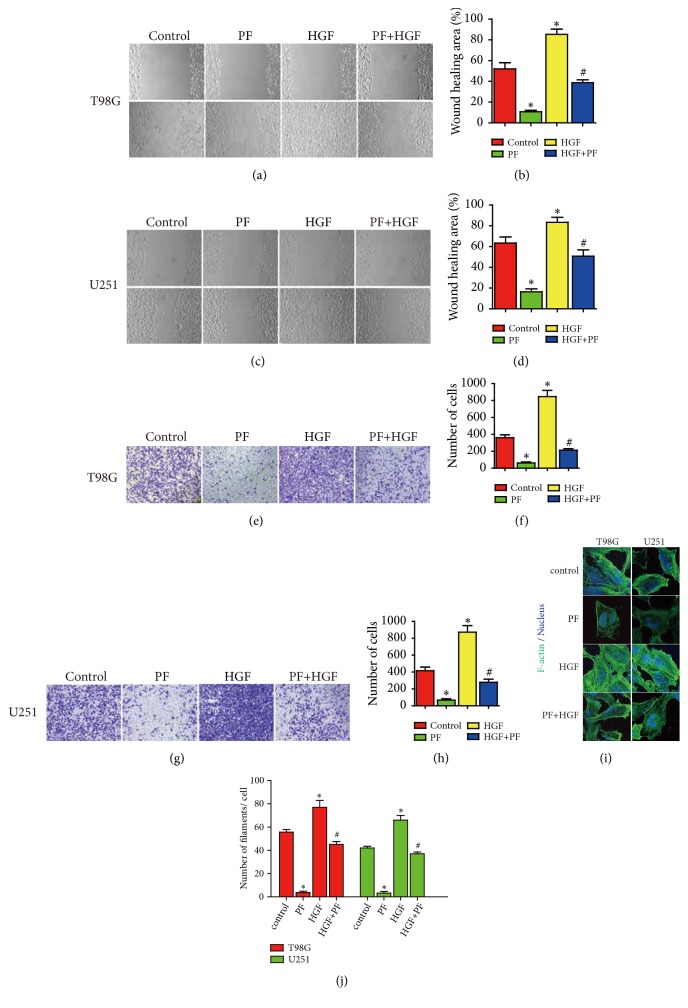
*Paeoniflorin inhibited HGF-mediated cell migration, invasion, and actin cytoskeleton arrangement in T98G and U251*. (a-d) Paeoniflorin inhibited HGF-induced cell migration. (e-h) Paeoniflorin inhibited HGF-induced cell invasion. (i, j) Paeoniflorin inhibited HGF-induced cell actin cytoskeleton arrangement. Control: no treatment; PF: 10*μ*M paeoniflorin treatment for 24h. HGF: 20ng/ml HGF treatment for 24h; PF+HGF: 20ng/ml HGF combined with 10*μ*M paeoniflorin incubation for 24h. All tests were performed in triplicate and presented as mean ± standard error. *∗*P < 0.05, compared with control group. ^#^P < 0.05, compared with HGF group.

To investigate whether paeoniflorin could suppress HGF-mediated migration and invasion, U251 and T98G cells were treated with HGF in the presence or absence of paeoniflorin. In Figures 3(a)-3(h), similarly, paeoniflorin significantly inhibited migration and invasion of U251 and T98G cells. Meanwhile, treatment with HGF significantly increased the ability of migration and invasion in U251 and T98G. Moreover, treatment with paeoniflorin combined with HGF decreased HGF-induced increasing of migration and invasion in both U251 and T98G.

Also, it has been reported that HGF could regulate actin cytoskeleton arrangement. To demonstrate this and investigate whether paeoniflorin could affect HGF-induced actin cytoskeleton arrangement, we detected F-actin pattern of U251 and T98G cells incubation with HGF with or without of paeoniflorin. In Figures 3(i)-3(j), paeoniflorin significantly induced actin cytoskeleton rearrangement that the normal actin filaments structure began to become disordered and sparse in U251 and T98G cells. At the same time, treatment with HGF make F-actin arrangement becomes denser and has more bundles compared with the control group in U251 and T98G. Moreover, incubation with paeoniflorin in combination with HGF reduced HGF-induced F-actin arrangement in both U251 and T98G.

**Figure 4:**
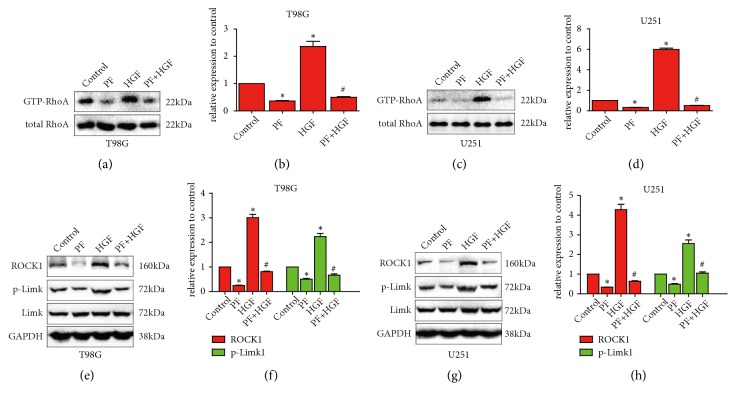
*Paeoniflorin inhibited HGF-induced RhoA/ROCK signaling activity*. T98G and U251 cells were pretreated with or without 10*μ*M paeoniflorin and after that the cells were stimulated with 20ng/ml HGF for 6 hours; then activity of GTP-RhoA (a-d) and the protein expression of ROCK1, Limk1, and p-Limk1 (e-h) were detected. Control: no treatment; PF: 10*μ*M paeoniflorin treatment for 24h. HGF: 20ng/ml HGF treatment for 24h; PF+HGF: 20ng/ml HGF combined with 10*μ*M paeoniflorin incubation for 24h. All tests were performed in triplicate and presented as mean ± standard error. *∗*P < 0.05, compared with control group. ^#^P < 0.05, compared with HGF group.

To further verify the effects of paeoniflorin on HGF-mediated migration, invasion, and actin rearrangement, we detected RhoA activity and expression of ROCK1 and p-Limk1; we found that HGF strengthen RhoA activity and expression of ROCK1 and p-Limk1 and paeoniflorin reversed the HGF-induced changes of RhoA activity and expression of ROCK1 and p-Limk1 in U251 and T98G (Figure 4). These consist with the results we obtained in migration, invasion, and actin rearrangement.

Taken together, paeoniflorin repressed HGF-mediated glioblastoma cells migration and invasion and caused actin rearrangement.

### 3.5. Paeoniflorin Suppressed HGF-Mediated Glioblastoma Cells Migration and Invasion and Induced Actin Rearrangement via Modulation of c-Met

**Figure 5:**
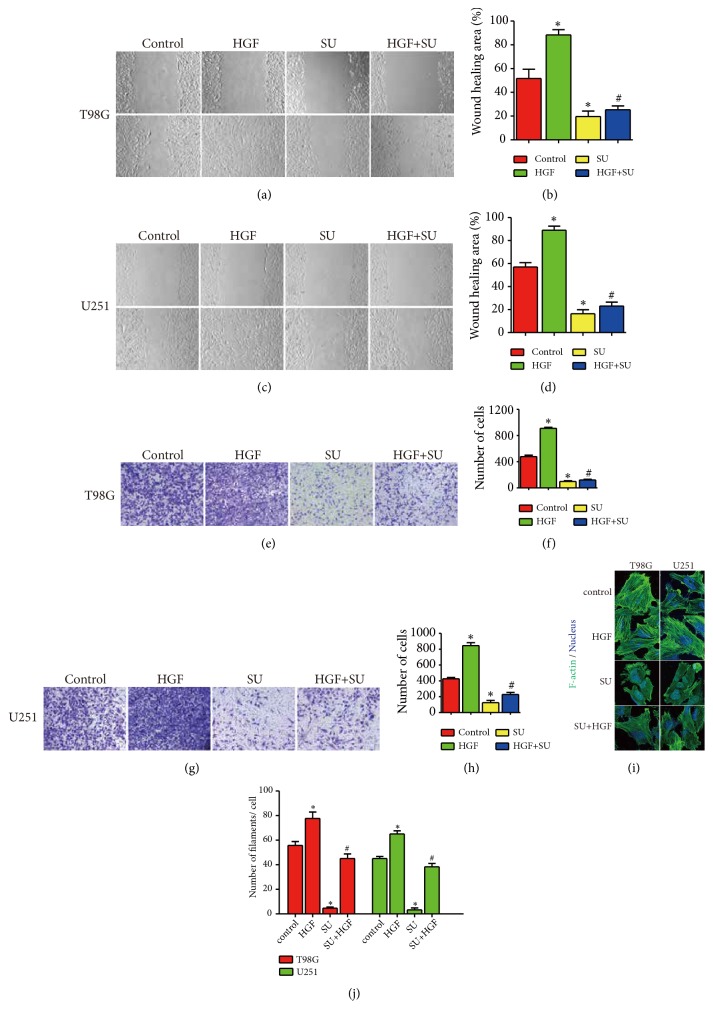
*SU11274 suppressed HGF-mediated migration, invasion, and actin cytoskeleton arrangement* T98G and U251 cells were incubated with 20 ng/ml of HGF for 24 hours and c-Met inhibitors SU11274 (5 *μ*mol/l) was used 4 hours before HGF treatment. (a-d) SU11274 inhibited HGF-induced cell migration. (e-h) SU11274 inhibited HGF-induced cell invasion. (i-j) SU11274 inhibited HGF-induced cell actin cytoskeleton rearrangement. Control: no treatment; HGF: 20ng/ml HGF; SU: c-Met inhibitor SU11274 (5*μ*mol/l); HGF+SU: 20ng/ml HGF combined with 5*μ*mol/l SU11274 (5*μ*mol/l) treatment. All tests were performed in triplicate and presented as mean ± standard error. *∗*P < 0.05, compared with control group. ^#^P < 0.05, compared with HGF group.

**Figure 6:**
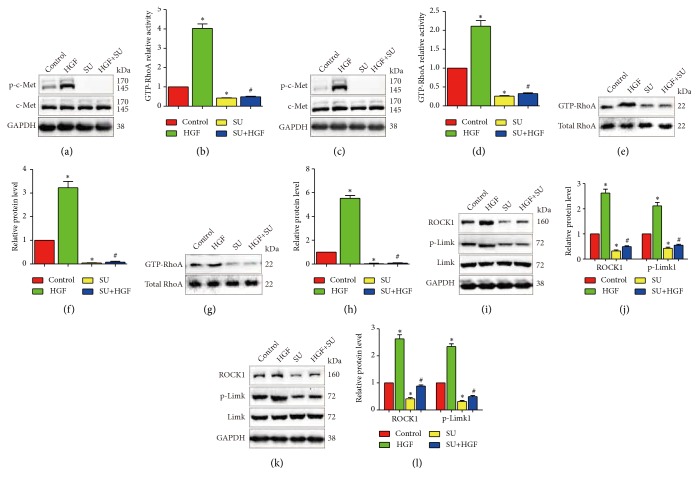
*SU11274 suppressed HGF-mediated RhoA/ROCK signaling activity in T98G and U251*. T98G and U251 cells were treated with 20 ng/ml of HGF for 15 minutes in present or absent of preincubation with SU11274 (5*μ*mol/l) for 6 hours, protein level of c-Met and p-c-Met (a-d), GTP-RhoA activity (e-h), protein expression of ROCK1, Limk1, and p-Limk1 (i-l) were detected by western blotting. Control: no treatment; HGF: 20ng/ml HGF; SU: c-Met inhibitor SU11274 (5*μ*mol/l); HGF+SU: 20ng/ml HGF combined with 5*μ*mol/l SU11274 (5*μ*mol/l) treatment. All tests were performed in triplicate and presented as mean ± standard error. *∗*P < 0.05, compared with control group. ^#^P < 0.05, compared with HGF group.

To test whether paeoniflorin inhibited HGF-mediated migration and invasion via the suppression of c-Met activation, U251 and T98G cells were treated with c-Met inhibitor SU11274. As shown in Figures 5(a)-5(h), U251 and T98G cells stimulated by HGF displayed more strong ability of migration and invasion compared with the control group. However, when these cells were treated with SU11274, the ability of migration and invasion induced by HGF was decreased. Similarly, the HGF-mediated intension of actin rearrangement can be abolished by SU11274 treatment (Figures 5(i)-5(j)). In parallel, the HGF-induced c-Met phosphorylation, changes of RhoA activity, and expression of ROCK1 and p-Limk1 expression were significantly upregulated, whereas SU11274 reversed HGF-induced upregulation of c-Met phosphorylation, RhoA activity, and expression of ROCK1 and p-Limk1 expression (Figure 6).

**Figure 7:**
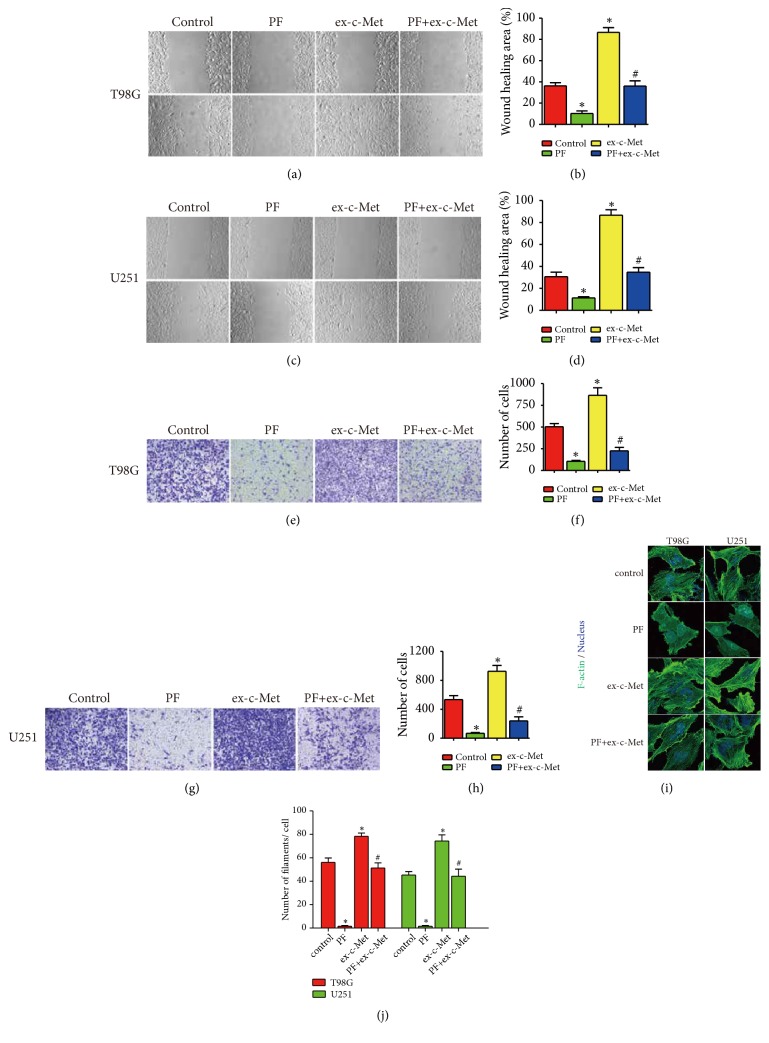
*Over-expression of c-Met reduced effects of paeoniflorin on cell migration, invasion,n and actin cytoskeleton rearrangement*. T98G and U251 were transfected vector plasmid or c-Met overexpression plasmid and treatment with or without 10*μ*M paeoniflorin; then the cell migration (a-d) and invasion (e-h) and actin cytoskeleton rearrangement and number (i-j) were evaluated. Control: vector plasmid; PF: 10*μ*M paeoniflorin +vector plasmid; ex-c-Met: c-Met overexpressed plasmid; PF+ex-c-Met: c-Met overexpressed plasmid +10*μ*M paeoniflorin. All tests were performed in triplicate and presented as mean ± standard error. *∗*P < 0.05, compared with control group. ^#^P < 0.05, compared with PF group.

**Figure 8:**
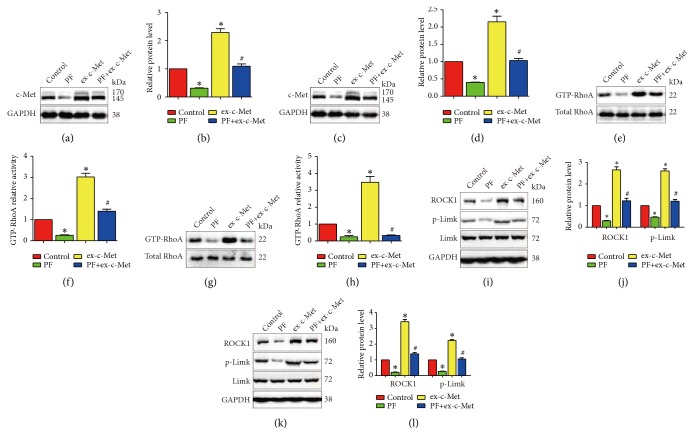
*Overexpression of c-Met reduced effects of paeoniflorin on c-Met/RhoA/Limk1 signaling*. T98G and U251 were transfected vector plasmid or c-Met overexpression plasmid and then treatment was with or without 10*μ*M paeoniflorin for 24h. The activity of GTP-RhoA (a-h) and the protein expression of ROCK1, Limk1, and p-Limk1 (i-l) were examined. Control: vector plasmid; PF: 10*μ*M paeoniflorin +vector plasmid; ex-c-Met: c-Met overexpressed plasmid; PF+ex-c-Met: c-Met overexpressed plasmid +10*μ*M paeoniflorin. All tests were performed in triplicate and presented as mean ± standard error. *∗*P < 0.05, compared with control group. ^#^P < 0.05, compared with PF group.

To further determine whether paeoniflorin suppressed glioblastoma cells migration, invasion, and actin rearrangement via c-Met, we transiently transfected U251 and T98G cells with c-Met overexpression plasmid (ex-Met). Compared to the control group, the ex-c-Met group showed more forceful migration and invasion ability as well as the more reinforced F-actin arrangement. Nevertheless, when treatment with paeoniflorin combined with c-Met overexpression, the expressed c-Met-induced intension of migration and invasion and F-actin arrangement were attenuated (Figure 7). Meanwhile, paeoniflorin significantly downregulated c-Met expression in U251 and T98G cells. In addition, overexpression ex-Met promoted RhoA activation and significantly upregulated expression of ROCK1 and p-Limk1 expression. At last, paeoniflorin significantly downregulated c-Met overexpression-induced RhoA activity and expression of ROCK1 and p-Limk1 (Figure 8). Taken together, the effects of paeoniflorin on HGF-mediated migration, invasion, and actin rearrangement are via modulation of HGF/c-Met.

## 4. Discussion 

An increasing number of evidences have demonstrated that HGF played a key role in a variety of cancer progressions and accelerated the tumor-promoting activity including glioblastoma. Paeoniflorin, as a natural polyphenolic product, showed effects of antiproliferative and anti-invasion activity on multiple tumors. In our present study, paeoniflorin suppressed HGF-mediated migration and invasion and actin cytoskeleton rearrangement of glioblastoma cells, and the mechanism involved c-Met/RhoA/ROCK1 signaling regulation.

Migration and invasion play an important role in various cancers progression which leads to the cancers infiltrate into the surround normal tissue even cause the distant metastasis. And cells migration and invasion involve multiple processes; among that the actin cytoskeleton rearrangement is a pivot one. The actin cytoskeleton rearrangement provides the engine during the process of cell motility [26]. So, regulating the rearrangement of actin cytoskeleton may lead to cancer cell migration and invasion suppression. In our study, paeoniflorin effectively inhibits migration and invasion in glioblastoma cells. Moreover, paeoniflorin treatment results in actin cytoskeleton reorganization, suggesting that paeoniflorin inhibits cell migration and invasion through regulation of actin cytoskeleton reorganization.

HGF is a multifunctional cytokine involved in the migration and invasion processes [27, 28]. Moreover, recent research has reported that paeoniflorin plays an important role in migration and invasion. For example, paeoniflorin suppressed invasion of breast cancer cells through affecting Notch-1 signaling pathway [14]. Paeoniflorin also repressed invasion in human hepatocellular carcinoma cells [8]. In our study, paeoniflorin suppressed HGF-mediated migration and invasion in U251 and T98G cells. Thus, we testify that paeoniflorin inhibits HGF-mediated migration and invasion in glioblastoma cells.

Rho-GTPase is the most relevant to actin cytoskeleton rearrangements and some research has validated that Rho-GTPase could be regulated by HGF/c-Met signaling [29, 30]. De Wever O et al. reported that SF/HGF enhanced human colon cancer cells invasion ability through RhoA and Rac1[31]. In addition, Takaishi K et al. demonstrated that HGF-induced cell motility involved Rho-GTPase regulation [32]. RhoA, one of Rho GTPase family members, is an intracellular molecular switch that transduces signals in various cancers and promotes actin polymerization. Our results showed that paeoniflorin suppressed HGF-induced RhoA activity as well as the downstream ROCK1 and Limk1 signaling. Our results suggest that paeoniflorin suppresses cell migration and invasion might be through regulating reorganization of the actin cytoskeleton via RhoA/ROCK signaling pathways.

The cell surface receptor tyrosine kinase c-Met is overexpressed in various cancers, including glioblastoma. c-Met acts as an important role in migration and invasion and associates with the poor prognosis in glioblastoma. c-Met can strengthen cell migration and invasion via a few pathways like the focal adhesion kinase (FAK), phosphatidylinositol 3-kinase (PI3K), and extracellular signal-regulated kinase (ERK) pathway [33–35]. Moreover, dysregulation of c-Met signaling could affect actin cytoskeleton rearrangement [36]. In our present research, HGF induced phosphorylation of c-Met and activates the RhoA/ROCK signaling in glioblastoma cells. What is more, paeoniflorin inhibited HGF-mediated phosphorylation of c-Met and the RhoA/ROCK activation. Furthermore, c-Met inhibitor SU11274 showed the similar effects to that of paeoniflorin, implying the key roles of c-Met/RhoA/ROCK signaling in the antitumor property of paeoniflorin.

To verify the property of paeoniflorin on migration, invasion, and actin cytoskeleton rearrangement in glioblastoma, we expressed c-Met by transfecting c-Met plasmid in U251 cells and T98G cells and found that c-Met promoted migration and invasion. The cells with high c-Met expression displayed denser F-actin filaments. GTP-RhoA expression was increased as well as its downstream signaling. Importantly, paeoniflorin could also suppress the migration and invasion and actin cytoskeleton rearrangement in overexpressed c-Met U251 cells and T98G cells, while GTP-RhoA was downregulated after paeoniflorin treatment. These results suggest that paeoniflorin reverse HGF-mediated migration, invasion, and actin cytoskeleton rearrangement by targeting c-Met and RhoA/ROCK pathway in glioblastoma cells.

In summary, our results demonstrated paeoniflorin inhibited HGF-mediated migration, invasion, and actin cytoskeleton rearrangement in glioblastoma cells. The mechanisms involved inhibition of c-Met/RhoA/ROCK signaling. This study provides an important basis for paeoniflorin application to treat glioblastoma.

## Data Availability

The data used to support the findings of this study are available from the corresponding author upon request.
